# Lipid bound extended release buprenorphine (high and low doses) and sustained release buprenorphine effectively attenuate post‐operative hypersensitivity in an incisional pain model in mice (*Mus musculus*)

**DOI:** 10.1002/ame2.12157

**Published:** 2021-03-23

**Authors:** Kaela Navarro, Katechan Jampachaisri, Monika Huss, Cholawat Pacharinsak

**Affiliations:** ^1^ Department of Comparative Medicine Stanford University Stanford CA USA; ^2^ Department of Mathematics Naresuan University Phitsanulok Thailand

**Keywords:** analgesia, analgesics, buprenorphine, mouse, pain management

## Abstract

**Background:**

Extended‐release buprenorphine (XR) is indicated for pain management in rodents, but little is known about its use in mice. This study aimed to investigate whether high dose XR effectively attenuates post‐operative hypersensitivity better than low dose XR in a mouse model of incisional pain.

**Methods:**

Mice (n = 44) were randomly assigned to 1 of 4 treatment groups: (a) saline (1 ml/kg SC, once); (b) sustained release buprenorphine (Bup‐SR, 1 mg/kg SC, once); (c) low dose extended‐release buprenorphine (XR‐lo, 3.25 mg/kg SC, once); (d) high dose extended‐release buprenorphine (XR‐hi, 6.5 mg/kg SC, once). On days −1, 0 (4 hours), 1, 2, and 3, mechanical and thermal hypersensitivities were evaluated, and plasma buprenorphine concentrations were measured.

**Results:**

Mechanical (days 0‐2) and thermal (days 0‐1) hypersensitivities were observed in the saline group. Bup‐SR, XR‐lo, and XR‐hi attenuated mechanical hypersensitivity on days 0, 1, and 2. None of the treatment groups, except XR‐Lo on day 0, attenuated thermal hypersensitivity on days 0 or 1. Plasma buprenorphine concentration peaked at 4 hours (day 0) in all treatment groups and remained greater than 1 ng/mL on days 0‐2. No abnormal clinical observations or gross pathologic findings were seen in any groups.

**Conclusion:**

The results indicate XR‐hi did not effectively attenuate post‐operative hypersensitivity better than XR‐lo. Thus both 3.25 and 6.5 mg/kg XR are recommended for attenuating post‐operative hypersensitivity for at least up to 48 hours in mice.

## 1 INTRODUCTION

Effective control of post‐operative pain is an important aspect for animal welfare^1^ and in conducting reproducible studies.^2^ Multiple studies^3^, ^4^, ^5^ have demonstrated post‐operative pain control techniques that decrease stress associated with frequent handling and frequent dosing. Methods include mixing analgesics in gel‐based oral compounds,^3^ drinking water,^6^, ^7^ food pellets,^4^ or via transdermal patches.^8^, ^9^ One common method is utilizing sustained release injectable analgesics. Sustained release buprenorphine (Bup‐SR) is a partial mu opioid receptor agonist popular in the research community for its long‐lasting analgesia. Bup‐SR has a high therapeutic index,^10^, ^11^ similar to buprenorphine‐HCl, and has been shown to provide analgesia in acute and chronic models of pain.^12^ Bup‐SR is reported to maintain therapeutic plasma concentrations for up to 48 hours in mice^13^ and 72 hours in rats.^14^ It has been reported to provide analgesia in a variety of species,^14^, ^15^, ^16^, ^17^, ^18^, ^19^, ^20^, ^21^ including mice for up to 12 hours^16^ and rats for 3‐5 days.^3^, ^14^, ^17^ While Bup‐SR has provided an effective option for managing post‐operative pain in rodents negative side effects have been noted, including pica,^22^ respiratory depression,^23^ sedation,^17^ mild skin lesions at the injection site,^11^, ^14^, ^16^ decreased water consumption,^6^, ^24^ and weight loss.^25^

Recently, an alternative formulation of long acting buprenorphine, extended release buprenorphine (XR), has been introduced for use in laboratory rodents. It is marketed to maintain clinically significant plasma concentrations in mice and rats for up to 72 hours after a single subcutaneous injection.^22^ However, little is known about its efficacy for rodents in the post‐operative period. The aim of this current study was to determine whether a high dose of XR (6.5 mg/kg) would attenuate mechanical and thermal hypersensitivities more effectively than a low dose of XR (3.25 mg/kg, the manufacturer's recommended dose) in a mouse plantar incisional pain model during the post‐operative period. We hypothesized that a high dose of XR would attenuate mechanical and thermal hypersensitivity more effectively than a low dose of XR in this model.

## 2 METHODS

### 2.1 Animals

Adult male C57BL/6 mice (*Mus musculus*; n = 44; weight, 28 ± 1.5 g; The Jackson Laboratory, Bar Harbor, ME) were utilized. Sentinel animals were free of minute virus of mice, mouse hepatitis virus, mouse rotavirus, Theiler's murine encephalomyelitis virus, Sendai virus, murine adenovirus 1 and 2, ectromelia virus, lymphocytic choriomeningitis virus, pneumonia virus of mice, respiratory enteric virus 3 (Reovirus 3), *Mycoplasma pulmonis*, endo‐ and ectoparasites, and pinworms. Mice were group housed in IVC caging (Innovive, San Diego, CA) on pre‐filled corncob bedding, fed a commercial diet (Teklad Global 18% Protein Rodent Diet 2018, Harlan Laboratories, Madison, WI), provided with acidified bottled water (Innovive, San Diego, CA) and Enviro‐dri (Lab Supply, Fort Worth, TX) for bedding and enrichment. Rooms were maintained on a 12:12‐hour dark:light cycle at 68‐79°F (20‐26°C) and 30‐70% relative humidity. Experiments were approved by Stanford University's Administrative Panel for Laboratory Animal Care. All mice were treated in accordance with the *Guide for the Care and Use of Laboratory Animals*.^1^ Mice were acclimated to the facility for a minimum of 3 days prior to baseline testing. Animals were weighed daily from 3 days prior to surgery until euthanasia. At the study's conclusion, animals were euthanized by carbon dioxide asphyxiation, followed by cervical dislocation.

### 2.2 Surgery

Mice were anesthetically induced via 1%‐4% isoflurane with 100% O_2_ in an induction chamber, and anesthesia was maintained using 0.8%‐3% isoflurane delivered via a nose cone. Sterile ophthalmic ointment was administered prior to surgery and animals were placed on a circulating warm‐water blanket. Cefazolin (20 mg/kg SC; GlaxoSmithKline, Research Triangle Park, NC) and warmed 0.9% saline (1 mL/kg) were administered prior to surgery once subcutaneously between the shoulders 5 minutes prior to skin incision, and the animals were then placed in ventral recumbency. The plantar surface of the left (ipsilateral) hindpaw was aseptically prepped. The surgery was adapted from a previously described incisional pain model for mice.^26^ Three millimeters from the tibiotarsal joint, a 5 mm longitudinal incision through skin and fascia was made on the midline on the plantar surface of the foot, extending towards the digits. The underlying muscle bundle was elevated using curved forceps, and a stab incision was made into the muscle with the point of a 15 blade without disrupting muscle attachments or underlying structures. Fine tipped forceps were then inserted into the incision and used to distract the muscle horizontally for 10 seconds. Saline (0.9%) was applied to the tissues and blotted for excess fluid. The skin was closed with a single horizontal mattress using 4‐0 silk suture. Triple antibiotic ointment (Johnson & Johnson, New Brunswick, NJ) was applied after closure. Mice were left to recover in a clean cage placed over a heating pad and monitored continuously during recovery. They were returned to the home cage once fully ambulatory.

### 2.3 Study design

Mice were randomly assigned to 1 of 4 treatment groups: saline (saline; n = 8; 5 mL/kg SC; 0.9% NaCl, Hospira, Lake Forest, IL); sustained release buprenorphine (Bup‐SR; n = 12; 1 mg/kg SC; buprenorphine SR‐LAB, 0.5mg/ml, Zoopharm, Fort Collins, CO); low dose extended release buprenorphine (XR‐Lo; n = 12; 3.25 mg/kg SC; Ethiqa^®^, 1.3 mg/mL, Fidelis, North Brunswick, NJ); high dose extended release buprenorphine (XR‐Hi; n = 12; 6.5 mg/kg SC). Saline and Bup‐SR were administered using a 25G needle. XR‐Lo and XR‐Hi treatments were administered using a 22G needle. All treatments were administered once subcutaneously over the left shoulder 1‐2 minutes prior to skin incision. After complete injection, digital pressure was applied to the injection site for 5 seconds to prevent leakage.

### 2.4 Behavioral assessment

Mice were acclimated to the facility for a minimum of 3 days prior to testing, and to the behavior testing room for 15 minutes prior to daily testing. Behavioral testing for mechanical and thermal hypersensitivities was conducted daily for pre‐surgery (D−1) and post‐surgery days (D0 (4 hours), D1, D2, and D3) between 0645 and 1100.

### 2.5 Mechanical hypersensitivity testing

To assess the response to mechanical stimuli, each mouse was placed in a clear acrylic enclosure (10.1 × 10.1 × 12.5 cm) on an elevated mesh stand (Electronic von Frey Mesh Stand, IITC Life Science, Woodland Hills, CA) with 0.64‐cm ‘waffle’ holes. Mice were acclimated to the testing enclosure for a minimum of 15 minutes before applying von Frey monofilaments with calibrated bending forces (0.6 g, Aesthesio, DanMic Global, San Jose, CA) for 10 trials. Each mechanical stimulus was applied for 1 second on various locations of the plantar surface on both hindpaws. Withdrawal responses were defined as a mouse lifting the paw off the mesh after von Frey stimulation during 10 applications of the monofilament. Mechanical hypersensitivity was defined as a significant increase in paw withdrawal frequency. The right (contralateral) hindpaw of each mouse served as a control. Mechanical hypersensitivity testing was performed prior to thermal hypersensitivity testing.

### 2.6 Thermal hypersensitivity testing

To assess the response to thermal stimuli, each mouse was placed in a clear acrylic enclosure (10.1 × 10.1 × 12.5 cm) on a tempered‐glass surface preheated to 29‐30°C (Plantar Analgesia Meter, IITC LifeScience). Mice were acclimated to the testing enclosure for a minimum of 15 minutes prior to focal (4 × 6 mm) radiant heat from a 50‐W light bulb with a beam intensity of 20% directed to the plantar surface of each hindpaw per trial. A cutoff of 20 s was set to prevent tissue injury. Each hindpaw was tested 4 times, with a minimum of 2 minutes between each trial. The mean of the last 3 trials was used to set the withdrawal latency (thermal latency). Thermal hypersensitivity was defined as a significant decrease in time to thermal latency. The right (contralateral) hindpaw of each mouse served as a control.

### 2.7 Plasma drug concentration analysis

Animals were assigned to the same treatment groups as the surgery portion of this experiment. Animals were induced with 1%‐4% isoflurane and injected with either saline (n = 2); Bup‐SR (n = 12); XR‐Lo (n = 16); XR‐Hi (n = 16), and left to recover in a warm recovery cage. Animals were euthanized as described under the plasma collection section at D0 (4 hours), D1, D2, and D3 for whole blood collection.

### 2.8 Plasma collection

Animals were induced with 3%‐4% isoflurane delivered in 100% oxygen and exsanguinated via retroorbital collection, followed by cervical dislocation. Whole blood was collected in lithium‐heparinized microtainers and spun in a microcentrifuge at 3451 g for 20 minutes. The plasma was separated, placed into cryogenic tubes, and stored in −80°C prior to shipment for analysis.

### 2.9 Plasma concentration analysis

Plasma samples were shipped overnight on dry ice to the McWhorter School of Pharmacy Pharmaceutical Sciences Research Institute (Samford University, Birmingham, AL) for evaluation of plasma buprenorphine concentrations by using liquid chromatography‐tandem mass spectrometry. Each sample had a minimum volume of 50 µL. Buprenorphine standard spiking solutions were prepared in 50:50 DI water:acetonitrile to give concentrations in plasma ranging from 0.2 to 200 ng/mL. The buprenorphine plasma samples and standards (100 μL) were fortified with internal standard (50 ng/mL terfenadine). Acetonitrile (1 mL) was added to precipitate the plasma proteins, and the mixture was vortexed and centrifuged. The organic layer was transferred to a clean test tube and evaporated to dryness under nitrogen in a water bath set at 50°C. The samples were reconstituted in dilution solvent and analyzed by HPLC MS/MS. Matrix matched standards and QC samples were prepared using blank control plasma.

### 2.10 Clinical observation and gross pathology

Clinical observation for abnormal behaviors (eg altered activity levels, mobility, ease of acclimation to testing environment) was performed daily. Gross pathology was performed at the end of the experiment.

### 2.11 Statistical analysis

To assess significance of differences in withdrawal responses by group and over time, two‐way repeated measures ANOVA with Bonferroni correction for multiple comparisons (R Development Core Team, 2015) was performed. Data were expressed as means ± SEM. Weights between days 1 and 3 were compared using paired t tests with one‐tailed test. A *P* value of less than 0.05 was considered significant.

## 3 RESULTS

### 3.1 Body weight

**FIGURE 1:**
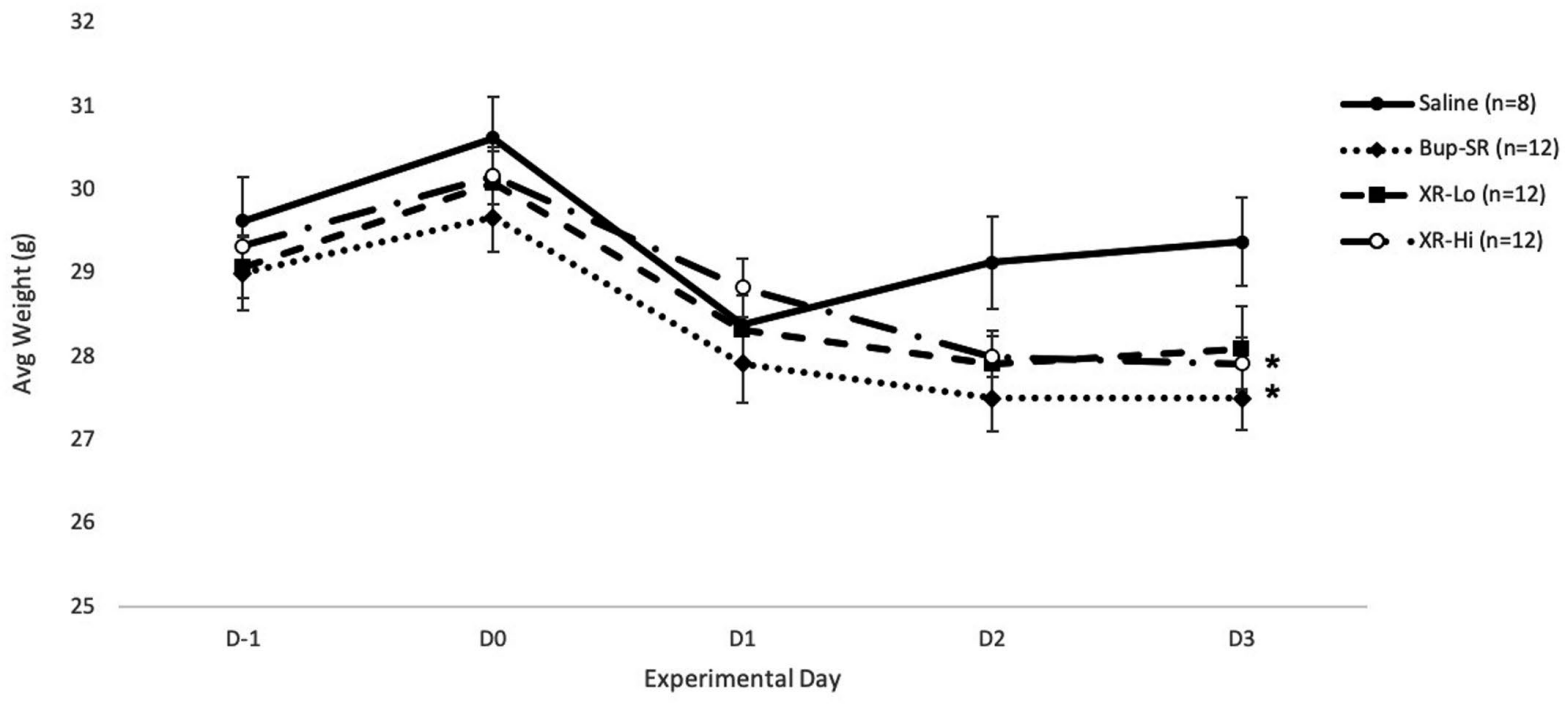
Body weights of mice over the course of the study. ^*^Significantly (*P* < .05) different on D3 vs baseline data (D−1)

Weight did not differ between groups prior to surgery (D−1). Weight in the saline and XR‐Lo groups did not differ significantly between D−1 (29.6 ± 0.5 g, 29.1 ± 0.4 g, respectively) and D3 (29.4 ± 0.5 g, 28.1 ± 0.5 g, respectively) (*P* = .563). Mice in Bup‐SR and XR‐Hi groups showed a significant decrease in weight from D−1 (29.0 ± 0.4 and 29.3 ± 0.3 g, respectively) to D3 (27.5 ± 0.4 (*P* = .005) and 27.9 ± 0.3 (*P* = .001) g), respectively (Figure 1 and Table 1).

**TABLE 1:** *p* values for body weights of mice (n = 12 per treatment group)

Treatment group	*p* value
Saline	.563
Bup‐SR	.005*
XR‐lo	.067
XR‐hi	.001*

* Day 3 value is significantly (*P* < .05) different from the day −1 value for the same treatment group.

### 3.2 Mechanical hypersensitivity

**FIGURE 2:**
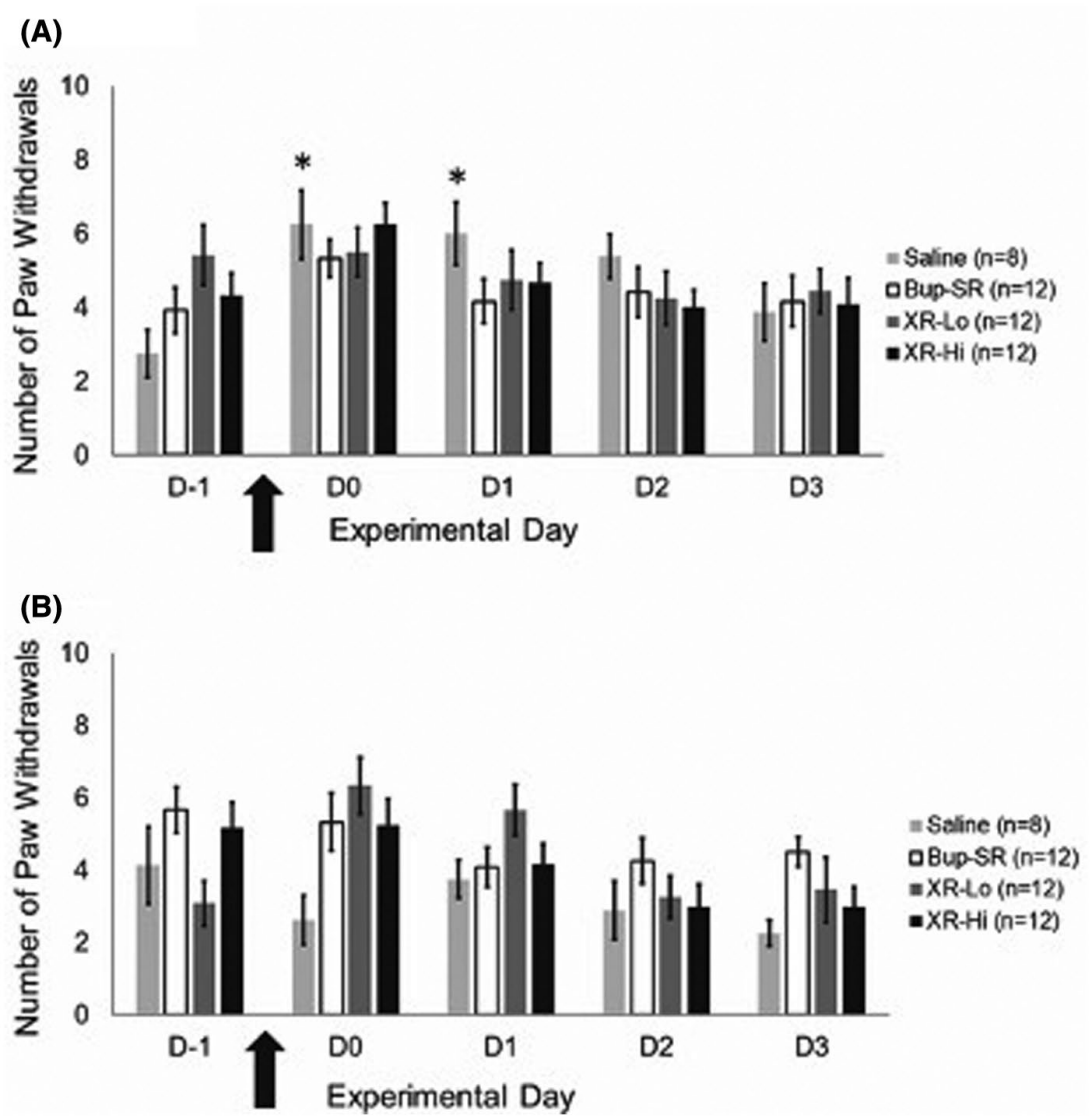
Mechanical hypersensitivity (number of paw withdrawals; mean ± SEM) of ipsilateral (A) and contralateral (B) paws. Arrow indicates the time of surgery. ^*^Significantly different (*P* < .05) from baseline (D−1) within the treatment group

In the ipsilateral hindpaw, mechanical hypersensitivity of the saline group was significantly increased only at D0 (4 hours, 6.3 ± 0.9 paw withdrawals; *P* = .001), D1 (6 ± 0.8 paw withdrawals; *P* = .000), and D2 (5.4 ± 0.6 paw withdrawals; *P* =.012) post‐surgery compared to D−1 (baseline value, 2.8 ± 0.6) (Figure 2A). Mechanical hypersensitivity of the Bup‐SR, XR‐Lo, and XR‐Hi groups was not significantly changed at any time point (D−1, D0, D1, D2, and D3). In the contralateral hindpaw, mechanical hypersensitivity of all groups did not differ from D−1 throughout the experiment (Figure 2B).

### 3.3 Thermal hypersensitivity

**FIGURE 3:**
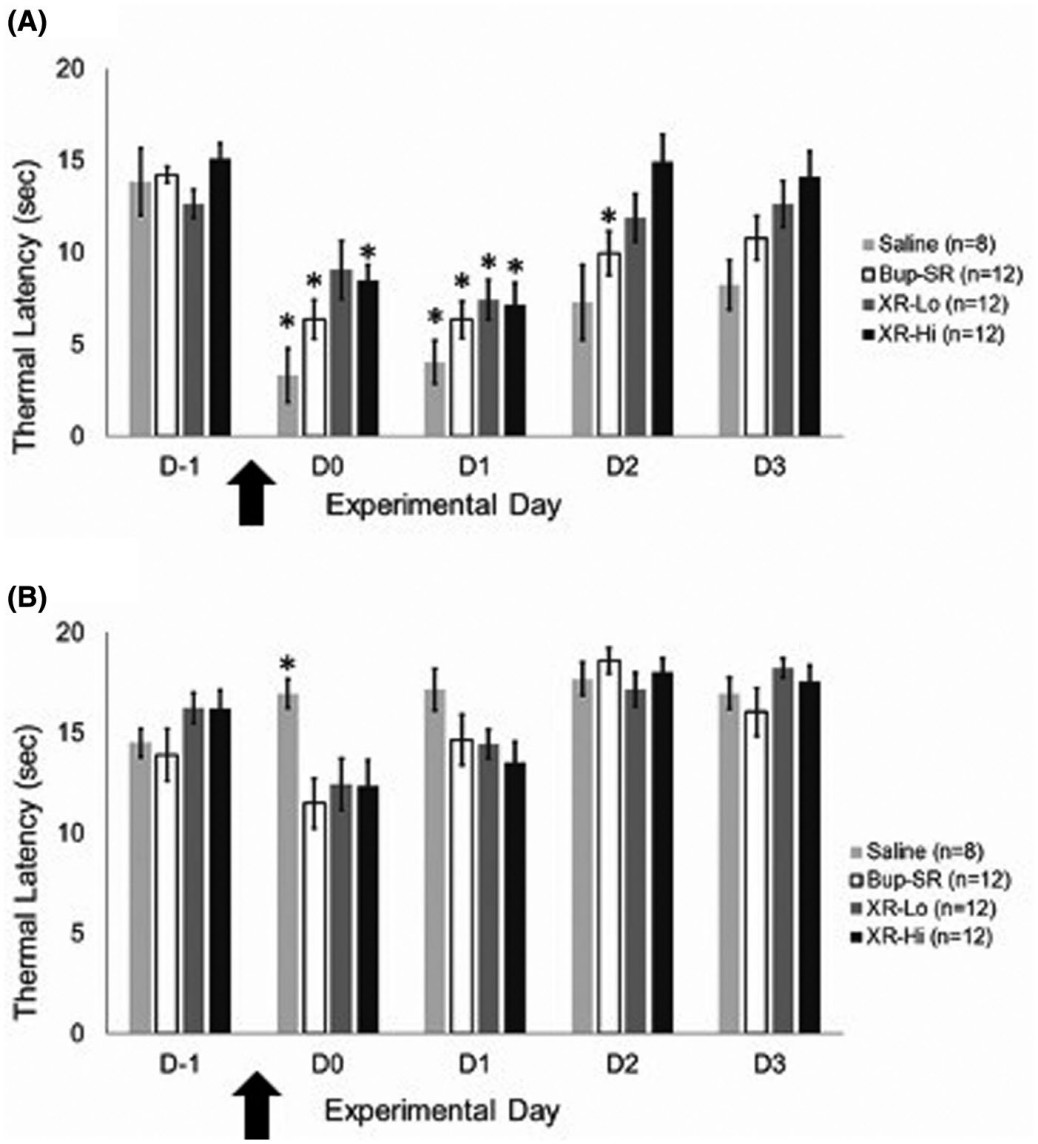
Thermal hypersensitivity (thermal latency; mean ± SEM) of ipsilateral (A) and contralateral (B) paws. Arrow indicates the time of surgery. ^*^Significantly different (*P* < .05) from baseline (D−1) within the treatment group

In the ipsilateral hindpaw, thermal hypersensitivity of the saline group was significantly increased at D0 (4 hours, 3.3 ± 1.5 seconds; *P* = .006) and D1 (4.0 ± 1.2 seconds; *P* = .006) post‐surgery compared to D−1 (baseline value, 13.8 ± 1.8 seconds) (Figure 3A). Thermal hypersensitivity of the Bup‐SR group was significantly increased on D0 (4 hours, 6.3 ± 1.1 seconds; *P* = .000), D1 (6.3 ± 1.0 seconds; *P* = .000), and D3 (10.8 ± 1.2 seconds; *P* = .004) compared to D−1 (14.2 ± 0.4 seconds). Thermal hypersensitivity of the XR‐Lo group did not differ on D0 (4 hours, 9.0 ± 1.6 seconds; *P* = .058) but was significantly increased on D1 (7.4 ± 1.1 seconds; *P* = .006) compared to D−1 (12.6 ± 0.8 seconds). In contrast, thermal hypersensitivity of the XR‐Hi group was significantly increased on D0 (8.4 ± 0.9 seconds; *P* = .000) and D1 (7.1 ± 1.2 seconds; *P* = .000) compared to D−1 (15.1 ± 0.8 seconds). In the contralateral hindpaw, except in the saline group on D0 (*P* = .000), thermal hypersensitivity of all groups did not differ from D−1 throughout the experiment (Figure 3B).

### 3.4 Plasma buprenorphine concentrations

**FIGURE 4:**
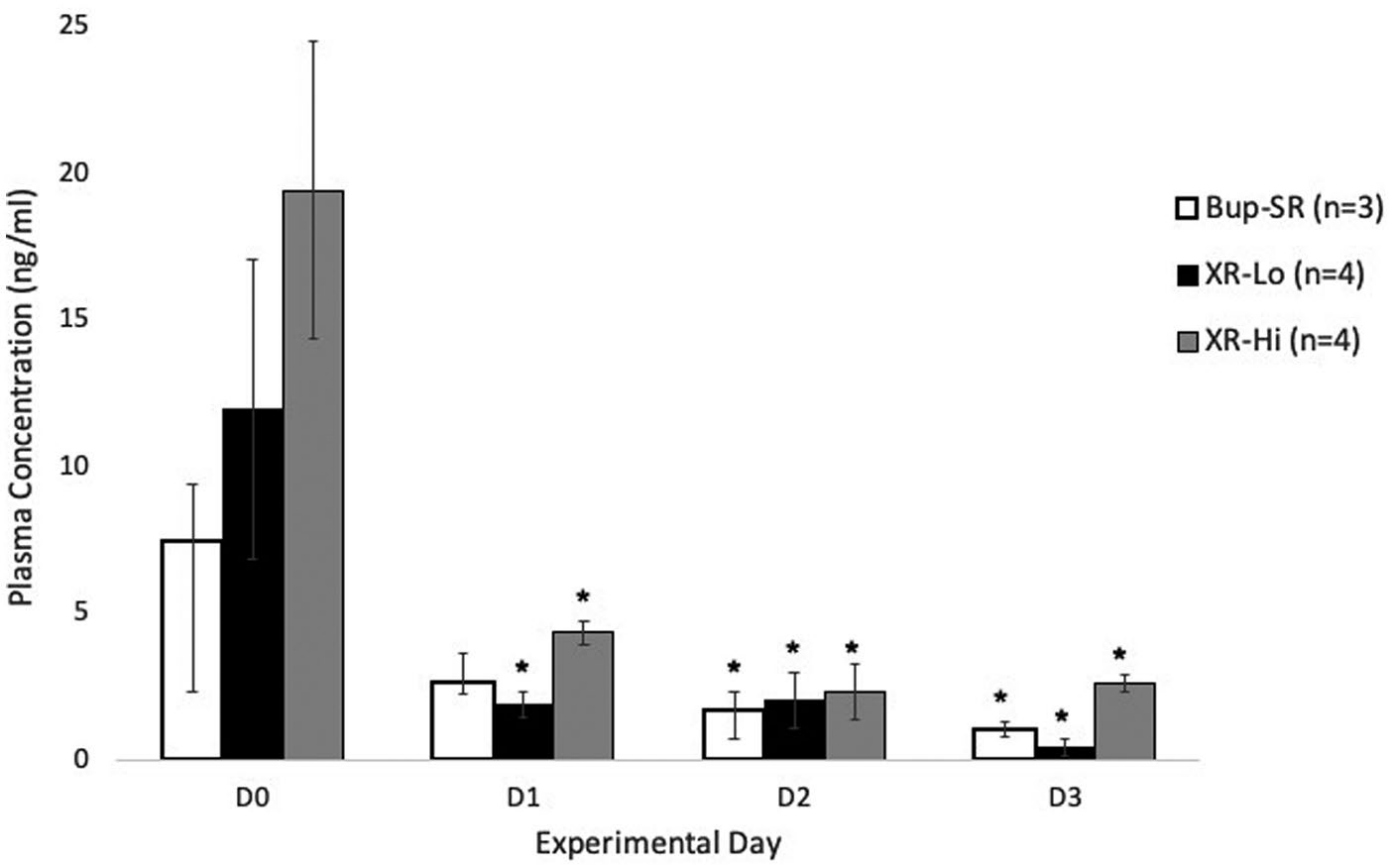
Plasma concentrations (ng/ml, mean ± SEM) of Bup‐SR, XR‐Lo, and XR‐Hi in treated mice (n = the number of animals sampled (3 or 4) at each time point). Samples were analyzed at 4 (D0), 24 (D1), 48 (D2), and 72 (D3) hours after administration. ^*^Significantly (*P* < .05) different from that on D0 within the same treatment group

Buprenorphine plasma concentrations remained above 1 ng/mL beginning at 4 hours after injection (D0; Bup‐SR = 7.4 ± 2.0 ng/mL, XR‐Lo = 11.9 ± 5.1 ng/mL, XR‐Hi = 19.4 ± 7.1 ng/mL) to D2 (Bup‐SR = 1.6 ± 0.6 ng/mL, XR‐Lo = 2.0 ± 1.0 ng/mL, XR‐Hi = 2.3 ± 0.9 ng/mL) (Figure 4). Plasma concentrations for buprenorphine were significantly decreased in the XR‐Lo (*P* = .014) and XR‐Hi (*P* = .034) group on D1 (Figure 4), but remained above 1 ng/mL (XR‐Lo = 1.9 ± 0.4 ng/mL, XR‐Hi = 4.3 ± 0.4 ng/mL). Plasma concentrations for both Bup‐SR and XR‐Hi remained at or above 1 ng/mL on D3 (Bup‐SR = 1.0 ± 0.2 ng/mL, XR‐Hi = 2.6 ± 0.9 ng/mL). The concentration for XR‐Lo on D3 was below 1 ng/mL (0.4 ± 0.3 ng/mL). Saline injected animals were used as negative controls on D0 (data not shown).

### 3.5 Clinical observation and gross pathology

Mice used in this study were more active and difficult to acclimate to the testing environment after opioid administration. The increased activity was observed at 4 hours (D0) and continued through 24 hours (D1) in all analgesic groups: Bup‐SR (83% and 17% of treated mice, respectively), XR‐Lo (83% and 67% of treated mice, respectively), and XR‐Hi (100% and 83% of treated mice, respectively). Gross pathologic examination performed at the end of the study revealed no gross abnormalities in any mice.

## 4 DISCUSSION

This study demonstrates that a single dose of XR‐Lo (3.25 mg/kg, SC) attenuates mechanical hypersensitivity as effectively as XR‐Hi (6.5 mg/kg, SC) for at least 1 day in a mouse incisional pain model. To our knowledge, this is the first study reporting the effects of buprenorphine extended release (XR) in a mouse model of incisional pain. No gross pathology was seen at necropsy. We recommend the use of Ethiqa‐XR at a dose of 3.25 mg/kg or 6.5 mg/kg for analgesic management of minor surgical procedures that use 1 mg/kg of Bup‐SR.

The aim of this study was to determine if a high dose of XR (6.5 mg/kg) would attenuate mechanical and thermal hypersensitivity more effectively than the manufacturer's recommended dose of 3.25 mg/kg using a mouse incisional model of acute minor pain. Our lab has extensive experience with the rat incisional pain model.^3^, ^4^, ^17^, ^27^, ^28^ Our previous studies have observed rat mechanical hypersensitivity lasting 1‐4 days^3^, ^27^, ^28^; other studies utilizing this model have seen rat mechanical hypersensitivity last up to 6 days.^4^, ^17^, ^27^, ^29^ The duration of mechanical hypersensitivity induced by this incisional model can vary by species^26^, ^29^ and strain,^30^, ^31^, ^32^ and was observed during thermal latency testing. Thermal hypersensitivity in this rat model has been seen to last 4 days.^3^, ^4^, ^27^, ^28^ Other groups using this model in mice observed mechanical (von Frey monofilament) and mouse thermal (Hargreaves method) hypersensitivity with an onset of 2 hours that lasted 2‐7 days.^26^, ^33^ In this study, the mouse incisional pain model caused an onset of mechanical hypersensitivity as early as 4 hours lasting up to D2 then returning to baseline levels. Although other groups have reported mouse thermal hypersensitivity lasting up to 7 days,^26^, ^33^ mouse thermal hypersensitivity returned to baseline levels by D2 in this study. These differences may be attributed to varying experimental conditions including strain,^32^ incision length (8 vs 5 mm), suture materials (staples, glue, nylon, vs silk), vendor (Jackson vs Charles River), and anesthetic regimens (phenobarbital vs isoflurane). Previous studies report that habituation time for mice is often 30 minutes or longer^34^, ^35^ whereas habituation time for rats is typically 5 minutes,^36^ which may also impact results. We observed that mouse thermal latency of the non‐surgical paws was significantly increased on D0 in the saline group but returned to baseline level by D1. This increased thermal latency in the non‐surgical paw may be reflective of delayed acclimation to the testing environment in our more active study mice.

Bup‐SR is frequently used in laboratory animals as it offers the benefit of a sustained duration of analgesia and requires less frequent dosing than buprenorphine HCl. Previous studies indicate that Bup‐SR attenuates post‐surgical pain for up to 1 day in mice undergoing laparotomies,^37^ and for 2‐3 days in a tibial defect rat model.^14^ In our study, Bup‐SR attenuated mechanical but not thermal hypersensitivity on days 0, 1, and 2. We expected thermal hypersensitivity would have been attenuated, as previous work from our group demonstrated that Bup‐SR attenuates both mechanical and thermal hypersensitivities for 5‐6 days in a rat plantar incisional model.^3^, ^17^ Other groups evaluating the efficacy of Bup‐SR in mice confirmed that Bup‐SR provides increased thermal latency time; however, these studies differed in that they were performed either in non‐surgical models,^5^, ^16^ or in surgical models where thermal hypersensitivity was not assessed in the incisional or peri‐incisional area.^37^ Our study evaluated thermal hypersensitivity in a surgical model which may be more painful than previous models used to evaluate thermal hypersensitivity in mice. It is known that the opioid dose required to attenuate thermal hypersensitivity can differ from the dose required to attenuate mechanical hypersensitivity.^38^, ^39^, ^40^ Therefore, it is possible the buprenorphine doses used here only effectively attenuated mechanical hypersensitivity. Unexpectedly, we saw a reduction in thermal latency on day 2 in the Bup‐SR group. It is possible that this is the result of an opioid‐induced hypersensitivity,^41^, ^42^, ^43^ which can be seen when low doses of opioids are used.

Sustained/extended release formulations of analgesics offer a method of refinement in laboratory animal husbandry, welfare, and analgesia. We sought to further refine and enhance the management of post‐operative pain by evaluating a new long‐lasting buprenorphine formulation (buprenorphine extended release, Ethiqa‐XR) post‐surgically. In this study, we found that both XR‐Lo and XR‐Hi effectively attenuated mechanical hypersensitivity with an onset as early as 4 hours post‐surgery, lasting up to 2 days. XR‐Lo, but not XR‐Hi, effectively attenuated thermal hypersensitivity at 4 hours (D0) post‐surgery. Buprenorphine's analgesic effect is reported to peak at 1 hours in humans^44^; Bup‐SR has been indicated to have analgesic effectiveness as early as 1^45^ to 2 hours after administration in mice.^16^ Similarly, the onset of analgesic effects for Ethiqa‐XR might be earlier than 4 hours, but should be confirmed by conducting hypersensitivity testing closer to administration. We decided to perform our testing at 4 hours post‐surgery to eliminate concerns of possible residual anesthetic effects.

In the present study, on D0 (4 hours after drug administration) all treatment groups achieved peak plasma buprenorphine concentrations, with XR‐Hi achieving the highest concentration (19.4 ng/mL). On D1, the XR‐Hi plasma buprenorphine concentration remained the highest (4.3 ng/mL) among all treatment groups. On D0, D1, and D2, mechanical hypersensitivity was attenuated in all treatment groups, with the lowest buprenorphine plasma concentration at 1.9 ng/mL (XR‐Lo, D1). These results support previously published reports of an effective therapeutic plasma concentration of buprenorphine being at least 1 ng/mL.^46^, ^47^, ^48^ In mice, plasma concentrations of Bup‐SR (1.0 mg/kg SC) were reported to be highest at 4 hours (14.5 ng/mL) after administration, decreasing to 4.2 ng/mL by 24 hours after administration.^13^ In this current study, plasma concentrations in all groups gradually declined over time. On D2 and D3, plasma concentrations were much lower (0.4‐2.5 ng/mL), with XR‐Hi achieving the highest plasma concentration. Therefore, based on the XR‐Hi plasma concentration observed in this study, XR‐Hi might effectively attenuate hypersensitivity on D3. The variability in the plasma concentrations reported over the course of this study may be attributed to analysis via mass spectrometry and the small sample size (3‐4 mice/group/time point) and this should be taken into consideration when evaluating these differences.

The difference in formulation technologies of XR compared to SR may contribute to the difference seen in hypersensitivity attenuation. Bup‐SR uses a liquid polymer dissolved in a biocompatible organic solvent.^49^ After injection, the buprenorphine encapsulated/entrapped within the liquid polymer is released over time as the polymer undergoes biodegradation via erosion of the polymer, hydrolysis, and drug diffusion.^50^, ^51^ In comparison, XR buprenorphine is bound within a lipid capsule and suspended in a medium chain fatty acid triglyceride which is degraded over time via lipase and esterase activity.^22^, ^52^ Differences in degradation may impact the rate of drug release, leading to different antinociceptive results. Additionally, other factors contributing to differences in our results could be the form of buprenorphine used (base or salt, in suspension or solution), or the types of vehicle (aqueous or oil‐based solution).^53^

In this current study, saline group body weights were not significantly different on D3 compared to D−1. For Bup‐SR and XR‐Hi groups, body weights were significantly reduced compared to baseline (D−1), though weight loss was less than 10% of baseline weights at all time periods. Buprenorphine administration has been reported to reduce weight^5^, ^12^, ^37^; however, some studies have shown weight gain after its use (Bup‐HCl at 0.2 mg/kg SC twice daily).^54^ The formulations of buprenorphine used here, sustained release (Bup‐SR) or extended release (XR), or the doses used may have more detrimental effects on weights. Additional side effects that have been reported after either buprenorphine or Bup‐SR administration are hyperactivity,^10^, ^55^, ^56^, ^57^, ^58^, ^59^ respiratory depression,^60^ abnormal behaviors, gastrointestinal tract motility,^5^ and disturbed circadian rhythm.^37^ Additionally, Bup‐SR (0.6 mg/kg^45^ and 1.5 mg/kg,^5^ SC) was reported to cause hyperactivity at 4 hours after administration, but not 24 or 48 hours in Swiss‐Webster mice.^5^ We observed hyperactivity on the day of drug administration in greater than 50% of opioid treated animals in each drug group, and at 24 hours post‐operatively in 17% of Bup‐SR animals, and greater than 50% of XR‐Lo and XR‐Hi animals. Hyperactivity may alter thermal latencies or mechanical hypersensitivity during testing which could have impacted our interpretation of thermal latency or mechanical testing results. Although buprenorphine‐induced hyperactivity is attributed to activation of μ opioid receptors,^58^ effects of XR’s delivery vehicle on hyperactivity cannot be ruled out and should be examined in future studies. Tidal volume was not measured in this study; however, respiratory depression was not clinically observed in any groups at any time point. In our previous studies assessing Bup‐SR in rats, sedation was noted after opioid administration,^3^, ^17^ which could explain why Bup‐SR showed thermal hypersensitivity attenuation in rats, but not mice.

Bup‐SR has also been seen to cause a variety of skin lesions^14^, ^16^ at the injection site in rodents. As discussed above, the delivery vehicles for Bup‐SR and XR differ, and thus skin reactions may differ. In the current study, we noted no erythema, ulceration, or inflammation of the skin at the injection site during or at the end of our study. Furthermore, the insert for Ethiqa‐XR notes that oily skin may be seen after administration. In this study, a 25 gauge needle was used to administer drugs and the skin was pinched after administration; no significant change in fur appearance was noted.

Opioid efficacy has previously been seen to differ in effectiveness among different mouse strains^32^, ^61^ and rat strains,^61^ and the impact of opioids on rodent immune function has been reviewed recently.^61^ Additionally, XR is a pharmaceutical grade FDA‐indexed analgesic, so its use will minimize the regulatory or voluntary accreditation issues that are associated with non‐pharmaceutical grade drugs.

The duration of Ethiqa‐XR‐induced analgesia should be further evaluated using a rodent model inducing long‐lasting pain, or in a more invasive pain model. The effect of mouse strain should also be further investigated, as strain can impact the ability of buprenorphine to alleviate hypersensitivity.^31^, ^32^ The effect of sex and age on Ethiqa‐XR’s analgesic efficacy should also be addressed in future studies. This additional work will better inform the lab animal community regarding the best post‐operative pain options to use in alignment with research objectives.

## AUTHORS’ CONTRIBUTIONS

K Navarro: 40%; K Jampachaisri: 15%; M Huss: 15%; C Pacharinsak: 30%.
